# A cross-sectional investigation into the occupational and socio-demographic characteristics of British police force employees reporting a dietary pattern associated with cardiometabolic risk: findings from the Airwave Health Monitoring Study

**DOI:** 10.1007/s00394-017-1562-4

**Published:** 2017-11-02

**Authors:** Rachel Gibson, Rebeca Eriksen, Deepa Singh, Anne-Claire Vergnaud, Andrew Heard, Queenie Chan, Paul Elliott, Gary Frost

**Affiliations:** 1 Nutrition and Dietetic Research Group, Faculty of Medicine, Imperial College, University of London, London, W12 0NN UK; 2 Department of Epidemiology and Biostatistics, MRC-PHE Centre for Environment and Health, School of Public Health, Imperial College, London, W2 1PG UK

**Keywords:** DASH (Dietary Approaches to Stop Hypertension Score), Cardiometabolic risk, Police, Diet

## Abstract

**Purpose:**

The aims of this study were to (1) determine the association between diet quality using the Dietary Approaches to Stop Hypertension (DASH) score and cardiometabolic risk in a British working population and (2) identify employee characteristics associated with reporting a poorer quality dietary pattern.

**Methods:**

British police employees enrolled (2007–2012) into the Airwave Health Monitoring Study (*n* = 5527) were included for sex-specific cross-sectional analyses. Dietary intakes were measured using 7-day food records. DASH score was calculated to determine diet quality. Logistic regression evaluated associations between (1) diet quality and increased cardiometabolic risk (defined as ≥ 3 risk markers: dyslipidaemia, elevated blood pressure, waist circumference, CRP or HbA1c), and (2) poor diet quality (lowest fifth of DASH score distribution) and employee characteristics.

**Results:**

Employees recording a poor diet quality had greater odds (OR) of increased cardiometabolic risk independent of established risk factors (demographic, lifestyle and occupational) and BMI: men OR 1.50 (95% CI 1.12–2.00), women: OR 1.84 (95% CI 1.19–2.97) compared to the healthiest diet group. Characteristics associated with reporting a poor quality diet were employment in Scotland vs. England: men OR 1.88 (95% CI 1.53–2.32), women: OR 1.49 (95% CI 1.11–2.00), longer working hours (≥ 49 vs. ≤40 h) men: OR 1.53 (95% CI 1.21–1.92) women: OR 1.53 (95% CI 1.12–2.09). For men, job strain (high vs. low) was associated with reporting a poor diet quality OR 1.66 (95% CI 1.30–2.12).

**Conclusions:**

The general population disparities in diet quality between England and Scotland were reflected in British police employees. The association of longer working hours and job strain with diet quality supports the targeting of workplace nutritional interventions.

**Electronic supplementary material:**

The online version of this article (doi:10.1007/s00394-017-1562-4) contains supplementary material, which is available to authorized users.

## Introduction

Employment is considered beneficial to health and well-being when occupational hazards are controlled [1]. Two types of work-related disease have been described by the World Health Organization: (1) ‘occupational diseases’ which occur as a direct result of workplace-specific exposures and (2) ‘multifactorial diseases’ which describe diseases that occur in the general population, but may be exacerbated or partially caused by workplace exposures [1]. The last few decades have produced an increasing number of studies suggesting that type two diabetes (T2DM) and cardiovascular disease (CVD) are multifactorial work-related diseases. Examples of occupational exposures associated with a disproportionate risk of poor cardiometabolic health include shift work [2], long working hours [3], and job strain [4].

It has been estimated that about a third of daily calorie intake is consumed in the workplace [5]. Therefore, diet should be an important consideration in the relationship between workplace exposures with cardiometabolic disease risk. The role of diet in the aetiology of cardiometabolic disease development and mortality is well established [6], with dietary modification central to the primary prevention and management of CVD and T2DM [7, 8].

The two foremost food-based dietary patterns that have shown to be beneficial in the management of cardiometabolic disease risk are the Mediterranean diet and the dietary approaches to stop hypertension diet (DASH) [9]. Dietary scores have been developed to measure dietary intake against these two dietary patterns in large cohort studies. Although both scores estimate a similar cardiometabolic disease risk reduction, it has been suggested that DASH captures the dietary characteristics related to T2DM to a greater extent than other diet quality scores [10]. Additionally, a meta-analysis of 20 randomised controlled trials of the DASH diet in the management metabolic risk found that in addition to a significant lowering of blood pressure, the DASH diet showed positive effects in the lowering of LDL cholesterol [11]. The DASH dietary pattern is characterised by high fruit, vegetable, low fat dairy and wholegrain intakes with low red meat, sugar-sweetened beverage and salt intakes [12].

To manage the increasing burden of cardiometabolic disease, public health strategies need to take account of wider environmental exposures as well as individual health behaviours. There is, therefore, a growing need to understand the relative influence of factors within different environments, such as the workplace, on dietary choice [13]. Research examining the relationship between occupational exposures and diet is currently lacking in large occupational groups in the UK.

The British police force employs over 250 000 men and women [14] across a range of job roles and geographical regions with varying working hour arrangements; however to date the diet, occupational, lifestyle and the cardiometabolic health of this large occupational group in the UK has not been studied. The Airwave Health Monitoring Study is a longitudinal study of British police force employees launched in 2004, with 42,112 participants enrolled at the end of 2012 [15]. Since April 2007, a sub-sample of participants completed a 7-day estimated weighted food diary (*n* = 15,404). The aims of this paper are to (1) measure the sex-specific association between diet quality determined Dietary Approaches to Stop Hypertension (DASH) score and cardiometabolic risk and (2) identify employee characteristics associated with reporting a dietary pattern associated with elevated cardiometabolic disease risk.

## Subjects and methods

### Participants

We conducted a cross-sectional study using baseline data collected as part of the Airwave Health Monitoring Study between 2007 and 2012. The Airwave Health Monitoring Study was open to all police forces in Great Britain, recruitment procedures and baseline characteristics have been described previously [15]. For the purpose of the present study, we included participants who had baseline dietary data available at the end of December 2015. We excluded participants with self-reported chronic disease diagnosis: angina, heart disease, angina, chronic obstructive pulmonary disease, cancer, chronic liver disease, thyroid disease and/or previous stroke (*n* = 322) as these diseases may affect cardiometabolic markers of interest. The present study sample (*n* = 5527) was comparable across key characteristics the total study sample (*n* = 42,112) [15]. The Airwave Health Monitoring Study is approved by the National Health Service Multi-site Research Ethics Committee (MREC/13/NW/0588).

### Assessment of dietary intake

Dietary intake was measured using 7-day estimated weighted food diaries. Participants were provided with written instructions and requested to provide details on cooking methods, brand names and portion sizes. To aid portion size estimation, photographs based on those developed by Nelson et al. were provided [16]. Calculation of nutritional intake was conducted using Dietplan6.7 software (Forestfield Software Ltd, Horsham, UK) which was based on the McCance and Widdowson’s 6th Edition Composition of Foods UK Nutritional Dataset (UKN). A team of trained coders ‘coded’ the diaries (matching of food and drink items recorded to a UKN database code and a portion size) follwing a study-specific standard protocol [17]. All coders were qualified nutritionists, dietitians or working towards a nutrition qualification. Mean energy intake (kcal/day) was derived from total energy intake divided by the number of complete food diary days. Energy-adjusted intakes (percentage contribution to total energy intake) were calculated for key macronutrient (total carbohydrate, non-milk extrinsic sugars, total fat, saturated fat, protein and alcohol). The Goldberg method was applied to estimate prevalence of energy intake misreporting [18]. The methodology applied and the results of estimated energy misreporting in this cohort have previously been reported in detail [17]. In the present study, we conducted sensitivity analyses to explore if energy intake misreporting among the Airwave Health Monitoring Study participants was a potential source of bias in the relationship between diet quality and cardiometabolic risk.

### Diet quality measurement

Mean daily intakes were calculated for ‘positive’ and ‘negative’ food groups (g/day) and sodium (mg/day) for each participant (positive food groups: Whole grains, low fat dairy, total fruit, vegetables excluding white potatoes, nuts, legumes and seeds; negative food groups: sugar-sweetened beverages, processed red meat and sodium) Supplementary Table S1 details the food group descriptions. The DASH scores were calculated as described by Fung et al. [12]. Participants were stratified by sex and the quintile (Q) of intake for each food group calculated using the following quintile score system. For positive food groups: Q1 = 1 point, Q2 = 2 points, Q3 = 3 points, Q4 = 4 points, Q5 = 5 points and for negative food groups reverse scoring was applied: Q1 = 5 points, Q2 = 4 points, Q3 = 3 points, Q4 = 2 points, Q5 = 1 point. The score ranges from 8 to 40, with a higher score thus corresponds to a more healthy diet pattern. Supplementary Table S2 shows the quintile cut-offs used to calculate the DASH score for the present study.

### Anthropometric, blood pressure and biochemical measurements

Enrolled participants attended a regional health-screening clinic. Trained research nurses used a standard protocol to conduct all clinical examinations as described previously [15]. Briefly, venous blood samples were drawn in the non-fasted state. Samples were processed on site and then transported (stored in a thermoporter at 0–4 °C) for processing at a designated study laboratory. Assays (HDL, non-HDL cholesterol, high sensitivity-C-reactive protein and HbA1c) were performed using serum, with the exception of HbA1c, which used whole EDTA blood samples (IL 650 analyser Instrumentation Laboratory, Bedford, Massachusetts, USA). Sitting blood pressure was measured using the Omron HEM 705-CP digital BP monitor (Omron Health Care). Three measurements were taken 30 s apart and the average recorded. Body weight was measured to the nearest 0.05 kg using digital scales (Marsden digital weighing scale). Standing height was measured to the nearest 0.1 cm (Marsden H226 portable stadiometer). Body mass index (BMI) was calculated as weight (kg)/weight (m^2^). Waist circumference was measured between the lower rib margin and the iliac crest in the mid-axillary line using a Wessex-finger/joint measure tape (Seca 201, Seca).

### Definition of cardiometabolic risk

For each participant, cardiometabolic risk was estimated based on the presence (yes/no) of five established risk parameters: (1) central adiposity (waist circumference ≥ 94 cm for men and ≥ 80 cm for women), (2) dyslipidaemia (HDL of < 1.0 mmol/L for men or < 1.3 mmol/L for women, and/or non-HDL ≥ 4.0 mmol/L, and/or prescribed lipid lowering medication), (3) elevated blood pressure (systolic ≥ 130 mmHg and/or diastolic ≥ 85 mmHg, and/or prescribed hypotensive medication), (4) inflammation (Hs-CRP ≥ 3 mg/L < 10 mg/L) and (5) impaired blood glucose control (HbA1c ≥ 5.7% and/or prescribed medication for glucose control). Increased cardiometabolic risk was defined as having three or more of these risk factors. Medication descriptions were matched to British National Formulary category and clinical indication determined.

### Employee characteristics

Information on occupational, lifestyle, medical history, socioeconomic and, demographic factors was collected during the health-screen visit using a structured on-line questionnaire. Total working hours (including usual weekly overtime) was classified into categories (< 40, 41 to 48, ≥ 49 h per week) based on previous research [3]. Job descriptors were collapsed from 31 to 2 categories of ‘work environment’ based on predominant working environment (mobile, or office based). Job strain dimensions were measured using a four-point Likert scale in response to six job content statements derived from the Karosek Job Content Questionnaire [19]. The quadrant approach was used to define job-strain [20]: high (low control, high demand), active (high control, high demand), passive (low, control, low demand), and low strain (high control, low demand). Physical activity information was collected using The International Physical Activity Questionnaire Short Form (IPAQ-SF) [21] which calculates metabolic equivalent (MET) minutes per week across three exercise domains (walking, moderate and vigorous) with participants categorised as undertaking a high, moderate or low level of activity [22]. Weekly TV viewing time was recorded as part of the lifestyle questionnaire in multiples of 15 min, and hours sitting per weekday were recorded in 5-h intervals. These variables were categorised into three groups (high, moderate and low) based on tertile cut-off values (TV viewing hours per week: low < 6, moderate 6 to 15, high > 15; weekly hours sitting on weekdays: low < 20, moderate 20 to 40, high > 40).

### Statistical analysis

All statistical analyses were undertaken using SAS version 9.3 (SAS Institute, Cary, NC, USA). To assess differences between two groups, independent *t* tests were used for data with a normal distribution (mean and standard deviation presented) and Mann–Whitney *U* tests were used otherwise (median and inter quartile range presented). Associations across categorical variables were analysed using Chi-Squared test (*χ* ^2^) with post-hoc comparisons conducted to determine the difference between subgroups [23]. To correct for multiple comparisons, the Bonferroni method was applied. As there were significant differences in occupational and lifestyle factors between men and women, we stratified all further analyses by sex. We used logistic regression to estimate the odds of having three or more markers of cardiometabolic risk per quintile of DASH score. The linear contrast function (SAS software) was used to test the presence of a linear trend (*P*_trend_) across the five ascending categories of DASH score (based on quintile ranges presented in Supplementary Table 3) and the association of having three or more cardiometabolic risk markers. Orthogonal polynomial coefficients (SAS function: PROC IML) were generated and applied to the contrast statement to correct for the unequal spacing between median values of each quintile of DASH score. Established confounding variables were included in the two models: (1) minimally adjusted: age (continuous), (2) fully adjusted: age plus categorical variables: physical activity, smoking status, education, TV viewing, job strain, menopause status (women only) and continuous variables: mean energy intake and mean alcohol g/day. Where a high level of collinearity was indicated between confounding variables (hours sitting and TV viewing, working hours and job strain) the variable explaining the greatest amount of variability was retained in the model. Due to the level of missing data for employment rank and work environment, these variables were not included in the multivariable analyses to maintain analytical sample size. Body mass index (BMI) was included in a separate model (continuous variable) as it potentially lies on the causal pathway between diet and markers of cardiometabolic health. Second, we used logistic regression to investigate the employee characteristics associated with consuming a poor quality diet (classified in the lowest DASH score group). Initially, we conducted bivariate logistic regression to determine the relationship between poor diet quality (yes, no) with each covariate. We then adjusted for established predictors of diet quality based on previous studies (age, smoking status, household income, physical activity, marital status, education, TV viewing, and country). Odds ratios (OR) are presented with corresponding 95% confidence intervals (95% CI).

## Results

### Population characteristics

The characteristics of the population sample by sex are shown in Table 1. From the 5527 participants included in the present study 60.3% were male, men were significantly older than women: 42.4 ± 8.9 vs. 39.5 ± 9.5 years, *p* < 0.0001. There were significant differences between men and women for all occupational characteristics measured, with men more likely to be in higher rank (8.8 vs. 2.6%, *p* < 0.0001), and have mobile job roles (58.7 vs. 45.1%, *p* < 0.0001) and work 49 h or more per week (26.2 vs. 12.5%, *p* < 0.0001). Women were more likely to be classified as having no markers of cardiometabolic risk compared to men (9.1 vs. 18.0%), while men were more likely to be classified as having three or more elevated risk markers (41.4 vs. 24.8%). Of those participants that had three or more risk factors, 84% men and 95% women, had central adiposity as one of the risk factors. Waist circumference was strongly correlated with BMI for men and women (Partial Pearson Correlation, age adjusted: men *r* = 0.84, *p* < 0.0001, women *r* = 0.88, *p* < 0.0001).

**Table 1:** Comparison of demographic, lifestyle and occupational characteristics across men and women in the Airwave Health Monitoring Study cohort (*n* 5527)

	Women		Men		P*
*N*, %	2195	39.7	3332	60.3	
Age, years, mean SD	39.5	9.5	42.4	8.9	< 0.0001
*N*, %					
White	2153	98.1	3222	96.8	
Relationship status					< 0.0001
Cohabiting	451	21.3	464	14.1	
Married	216	10.2	219	6.7	
Single	1046	49.5	2375	72.2	
Divorced/separated	401	19.0	230	7.0	
Missing	81		44		
Education					0.006
Left school before taking GCSE	74	3.4	155	4.7	
GCSE or equivalent	611	27.8	1023	30.7	
Vocational qualifications	160	7.3	240	7.2	
A levels / Highers or equivalent	718	32.7	1079	32.4	
Bachelor Degree or equivalent	477	21.7	637	19.1	
Postgraduate qualifications	155	7.1	197	5.9	
Annual household income					< 0.0001
Less than £32,000	580	26.4	298	8.9	
£32,000–£47,999	246	11.2	390	11.7	
£48,000–£57,999	698	31.8	1470	44.1	
£58,000–£77,999	428	19.5	849	25.5	
More than £ 78,000	243	11.1	324	9.7	
Employment (force) country					< 0.0001
England	1629	74.5	2299	69.2	
Scotland	310	14.2	647	19.5	
Wales	248	11.3	377	11.4	
Missing	8		9		
Rank					< 0.0001
Non-ranked police staff/other	1099	57.2	612	21.0	
Police Constable/ Sergeant	771	40.1	1983	68.2	
Inspector/Chief Inspector or above	50	2.6	255	8.8	
Other	3	0.2	59	2.0	
Missing	272	*12.0*	*423*	*12.7*	
Work environment					< 0.0001
Mainly office duties	689	54.9	998	41.3	
Mainly mobile duties	566	45.1	1417	58.7	
Missing/unclassified	940	42.8	917	27.5	
Total hours worked per week					< 0.0001
≤ 40 h	1387	63.2	1197	35.9	
41–48 h	533	24.3	1263	37.9	
≥ 49	275	12.5	872	26.2	
Job Strain					< 0.0001
Low (high control, low demand)	570	26.0	1079	32.4	
Passive (low control, low demand)	555	25.3	585	17.6	
Active (high demand, high control)	538	24.5	952	28.6	
High (high demand, low control) (N, %)	532	24.2	716	21.5	
Physical activity†					< 0.0001
Low	314	14.3	346	10.4	
Moderate	1061	48.3	1455	43.7	
High	820	37.4	1531	45.9	
Smoking status					< 0.0001
Never smoker	1489	68.1	2333	70.3	
Former smoker	472	21.6	753	22.7	
Current smoker	227	10.4	234	7.1	
Missing	17		2		
Sleep					< 0.0001
5 h or less	140	6.4	165	5.0	
6 h	471	21.5	1005	30.2	
7 h	903	41.1	1448	43.5	
8 h	586	26.7	624	18.7	
9 h or more	95	4.3	89	2.7	
Missing			1		
Sitting (total weekdays)					0.09
Low (< 20 h)	707	32.2	1023	30.7	
Moderate (20–40 h)	862	39.3	1267	38.0	
High (> 40 h)	626	28.5	1042	31.3	
Weekly TV viewing					< 0.0001
Low (< 6 h)	767	34.9	858	25.7	
Moderate (6–15 h)	963	43.9	1506	45.2	
High (> 15 h)	465	21.1	968	29.1	
Elevated cardiometabolic risk					
Waist circumference^a^	1130	51.5	1610	48.3	0.021
Dyslipidaemia^b^	509	23.2	1784	53.4	< 0.0001
Blood pressure^c^	735	33.5	2288	68.7	< 0.0001
Blood glucose^d^	963	43.9	1280	38.4	< 0.0001
Inflammation (CRP)^e^	357	16.3	355	10.7	< 0.0001
No. cardiometabolic risk markers					< 0.0001
Nil	395	18.0	302	9.1	
One	698	31.8	755	22.7	
Two	556	25.3	894	26.8	
Three	347	15.8	830	24.9	
Four	152	6.9	471	14.1	
Five	47	2.1	80	2.4	
Body mass index					< 0.0001
Healthy (< 25 kg/m^2^)	1110	50.6	721	21.6	
Over weight (25–30 kg/m^2^)	752	34.3	1845	55.4	
Obese (> 30 kg/m^2^)	333	15.2	766	23.0	

*SD* standard deviation, *GCSE* general certificate of Secondary Education, *EI* energy intake

*Student *t* test compared mean values between male and female participants. Chi-squared test compared differences between men and women for categorical variables, missing data was not included in the analyses, overall p value for Chi-square test presented. † METS metabolic equivalents, classification by IPAQ guidelines (21)

^a^Waist circumference: ≥94 cm (men) ≥ 80 cm (women);

^b^Dyslipidaemia HDL: <1.0 mmol/L (men); <1.3 mmol/L (women) and/or Non HDL ≥ 4 mmol/L and/or Reported diagnosed dyslipidaemia and/or self-reported lipid lowering medication

^c^Blood pressure Systolic ≥ 130 mmHg and/or diastolic ≥ 85 mmHg and/or self-reported diagnosed hypertension and/or taking hypotensive medication

^d^Blood glucose HbA1c ≥ 5.7% and/or self-reported diagnosed diabetes and/or taking medication for glucose control

^e^Inflammation C-reactive protein ≥ 3 mg/L < 10 mg/L, 39 participants had missing or CRP values above 10 mg/L

### Dietary descriptive statistics

There were significant differences between sources of energy intake between men and women. Women derived more energy from carbohydrates (48.0 ± 7.1 vs. 46.7 ± 6.9%, *p* < 0.0001) and non-milk extrinsic sugars (12.3 ± 5.2 vs. 11.6 ± 4.9%, *p* < 0.0001), while men obtained more energy from alcohol (4.4 IQR 7.6 vs. 2.9 IQR 6.6%, *p* < 0.0001). With the exception of whole grains and sodium, there were significant differences between energy-adjusted intakes across all food groups and dietary fibre, Table 2. Women consumed more, low fat dairy per 1000 kcal than men, while men consumed more red meat (including processed meat) per 1000 kcal. Women consumed diets with a higher concentration of fruit, vegetables, legumes, dairy and sugar-sweetened beverages (g/1000 kcal) compared to men.

**Table 2:** Dietary intakes of men and women in the Airwave Health Monitoring Study (*n* 5527)

	Women *(n* = 2352)		Men (*n* = 3497)		
DASH score, median (range)	24	9–38	24	9–39	

	Mean, SD				*P*
Mean daily energy intake, kcal	1674	386	2077	473	< 0.0001
Energy density food kcal/g	1.5	0.4	1.6	0.4	< 0.0001
% energy intake: total fat	33.8	5.7	33.5	5.4	0.08
% energy intake: saturated fat	12.4	2.9	12.3	2.8	0.67
% energy intake: protein	17.0	3.4	17.2	3.4	0.028
% energy intake: carbohydrate	48.0	7.1	46.7	6.9	< 0.0001
% energy intake NMEs	12.3	5.2	11.6	4.9	< 0.0001

	Median, IQR				
% energy intake: alcohol^a^	2.9	6.6	4.4	7.6	< 0.0001
NSP g/1000 kcal	7.1	2.9	6.6	2.7	< 0.0001
DASH score food group intakes					
Fruit g/1000 kcal	77.7	95.5	65.7	85.6	< 0.0001
Vegetables g/1000 kcal	79.7	57.5	59.3	43.2	< 0.0001
Legumes g/1000 kcal	11.7	15.4	10.9	13.0	0.031
Whole grains g/1000 kcal	19.4	28.1	19.0	32.0	0.79
Low fat dairy g/1000 kcal	96.6	91.8	92.4	83.3	0.008
Red meat g/1000 kcal	49.5	46.7	74.1	54.4	< 0.0001
Sugar sweetened beverages g/1000 kcal	34.7	110.9	29.4	103.0	0.017
Sodium mg/1000 kcal/day	1449	407	14,60	373	0.24

	*N*, %				
Discretionary salt usage^b^					0.19
No salt added	999	52.9	1407	51.2	
Salt added either table or cooking	506	26.8	742	27.0	
Salt added both table and cooking	229	12.1	390	14.2	
Salt substitute used	155	8.2	208	7.6	

Student *t* test compared mean values between male and female participants, Mann–Whitney *U* test compared median values between male and female participants and Chi-squared test to compare differences between men and women across categorical variables

*IQR* interquartile range, *NME* non-milk extrinsic sugars, *NSP* non-starch polysaccharides (estimated from Englyst values).

^a^Includes non-consumers

^b^Salt questionnaire completed by 4636 (missing data not included in analyses)

### DASH score and cardiometabolic risk

Logistic regression analyses showed a dose–response relationship across quintiles of DASH score and the odds of having three or more markers of cardiometabolic risk for men and women (*p* trend < 0.0001), Table 3. The association remained for men and women in the lowest DASH score group with an OR of 1.84 (95% CI 1.43, 2.38) and 1.92 (95% CI 1.35, 2.84), respectively, for recording three or more cardiometabolic risk factors. There was an attenuation of the OR following further adjustment for BMI (men: OR 1.50, 95% CI 1.12, 2.00; women: OR 1.84, 95% CI 1.19, 2.97), however, the significant dose–response relationship across quintile groups of DASH score and three or more metabolic risk factors were maintained.

**Table 3:** Odds ratio of having three or more markers of metabolic risk per quintile of DASH score: men(*n* = 3278) and women (*n* = 2139)

	Cases/*N*	Minimal adjusted^b^		Fully adjusted^a^		Fully adjusted^a^ + BMI	
		OR	(95% CI)	OR	(95% CI)	OR	(95% CI)
Men							
Ref: Q5 (healthiest)	235/636	1.00		1.00		1.00	
Q4	265/658	1.24	(0.98, 1.57)	1.19	(0.93, 1.50)	1.10	(0.84, 1.44)
Q3	330/763	1.62	(1.29, 2.03)	1.54	(1.22, 1.92)	1.32	(1.02, 1.71)
Q2	271/640	1.72	(1.36, 2.19)	1.52	(1.19, 1.94)	1.22	(0.92, 1.60)
Q1 (unhealthiest)	256/581	2.16	(1.67, 2.77)	1.84	(1.43, 2.38)	1.50	(1.12, 2.00)
*p* trend		***< 0.0001***		< ***0.0001***		***0.006***	
Women							
Ref: Q5 (healthiest)	87/406	1.00		1.00		1.00	
Q4	107/444	1.39	(0.98, 1.95)	1.35	(0.92, 1.92)	1.35	(0.91, 2.02)
Q3	126/473	1.85	(1.32, 2.60)	1.82	(1.29, 2.58)	1.74	(1.17, 2.57)
Q2	108/438	1.84	(1.30, 2.61)	1.72	(1.21, 2.48)	1.57	(1.04, 2.35)
Q1 (unhealthiest)	103/378	2.21	(1.53, 3.19)	1.92	(1.35, 2.84)	1.84	(1.19, 2.97)
* p* trend		< ***0.0001***		***0.0003***		***0.005***	

*BMI* body mass index, *CI* confidence intervals, *OR* odds ratio

24 participants did not have Hs-CRP available for metabolic risk calculation. Total cases = 1357/3278

15 participants did not have Hs-CRP available for metabolic risk calculation. Total cases = 531/2139

^a^Fully adjusted: age + physical activity, smoking status, education and TV viewing, job strain, continuous variables: mean energy intake and mean alcohol g/day. Women additionally adjusted for menopause status

^b^Minimal adjusted = age at health screen

### Predictors of poor quality diet pattern

In bivariate logistic regression household income was associated with poor diet quality (lowest fifth of DASH score distribution) in men but not in women, though in men the negative association observed in the highest income brackets did not remain significant after adjustment for established predictors of diet quality (age, education, household income, marital status, geographical region, smoking and TV viewing). Advancing age was associated with reduced odds of having a poor diet as was being in the highest category for physical activity. The highest category of education (bachelor degree or higher) was negatively associated with poor diet quality, and remained significant after adjustment (bachelor degree or higher vs. GCSE or below: men: OR 0.53 95% CI 0.41, 0.70; women: OR 0.43 95% CI 0.31, 0.60). For men and women geographical region and smoking status were positively associated with poor diet quality (Scotland vs. England: men: OR 1.88 95% CI 1.53, 2.32; women: OR 1.49 95% CI 1.11, 2.00; current vs. never smoker men: OR 1.90 95% CI 1.41, 2.58, women: OR 3.35 95% CI 2.47, 4.55), Table 4. These associations remained significant after adjustment for established predictors of diet quality. High and passive job strain (vs. low) was associated with increased odds of having a poor diet for men, though after adjustment only high job strain reminded significant (OR 1.38 95% CI 1.06, 1.58). Working 49 h or more per week (compared to < 40 h) was associated with poorer diet quality in men and women (men OR 1.53 95% CI 1.21, 1.92; women: OR 1.53 95% CI 1.12, 2.09), this only remained significant for men after adjustment. For men, the trend across ascending groups of working hours and increased cardiometabolic risk was significant (*P*_trend_ = 0.037).

**Table 4:** Odds ratio (OR) of being in the lowest DASH diet quality group for men and women (*n* 5527)

	Women								Men								
	Unadjusted^a^				Adjusted^b^				Unadjusted^c^					Adjusted^d^			
	OR	(95% CI)		P*	OR	(95% CI)		P*	OR	(95% CI)			P*	OR	(95% CI)		P*
Age (per 5 year increase)	0.79	(0.74	0.82)		0.74	(0.68	0.80)		0.77	(0.73	0.82)			0.76	(0.71	0.81)	
Relationship status				–				–					–				–
Ref: Cohabiting	1.00				1.00				1.00					1.00			
Divorced/separated/other	0.56	(0.36	0.88)		0.83	(0.49	1.41)		0.71	(0.48	1.06)			1.00	(0.64	1.56)	
Married	0.62	(0.47	0.82)		0.91	(0.66	1.25)		0.58	(0.46	0.74)			0.77	(0.59	1.03)	
Single	1.09	(0.79	1.50)		1.09	(0.75	1.60)		1.17	(0.82	1.68)			1.17	(0.78	1.73)	
Education				0.828				0.312					0.025				0.208
Ref: GCSE or below	1.00				1.00				1.00					1.00			
Vocational qualifications	0.95	(0.67	1.47)		0.67	(0.41	1.08)		1.32	(0.94	1.84)			1.10	(0.77	1.56)	
A levels/highers or equivalent	0.92	(0.71	1.20)		0.68	(0.50	0.91)		1.07	(0.87	1.32)			0.83	(0.65	1.04)	
Bachelor degree or higher	0.68	(0.51	0.91)		0.43	(0.31	0.60)		0.74	(0.58	0.94)			0.53	(0.41	0.70)	
Annual household income				0.818				0.032					0.465				0.973
Ref: less than £32,000	1.00				1.00				1.00					1.00			
£32,000–£47,999	0.88	(0.60	1.29)		1.00	(0.65	1.53)		0.96	(0.66	1.40)			1.07	(0.71	1.61)	
£48,000–£57,999	0.91	(0.69	1.20)		1.03	(0.72	1.44)		0.95	(0.70	1.29)			1.07	(0.75	1.53)	
£58,000–£79,999	0.89	(0.64	1.22)		0.94	(0.63	1.43)		0.69	(0.49	0.97)			0.84	(0.58	1.23)	
More than £78,000	0.64	(0.42	0.97)		0.90	(0.55	1.46)		0.64	(0.42	0.98)			0.93	(0.59	1.48)	
Total hours worked per week				0.776				0.836					0.004				0.037
Ref: ≤ 40 h	1.00				1.00				1.00					1.00			
> 40 < 48 h	1.04	(0.80	1.35)		0.97	(0.72	1.30)		1.37	(1.11	1.70)			1.23	(0.97	1.53)	
> 49 h	1.53	(1.12	2.09)		1.33	(0.93	1.91)		1.53	(1.21	1.92)			1.36	(1.06	1.75)	
Employment (force) country				–				–					–				–
Ref: England	1.00				1.00				1.00					1.00			
Scotland	1.49	(1.11	2.00)		1.90	(1.38	2.63)		1.88	(1.53	2.32)			2.25	(1.80	2.81)	
Wales	1.04	(0.73	1.48)		1.00	(0.68	1.46)		1.11	(0.83	1.48)			1.24	(0.92	1.69)	
Rank				0.421				0.772					0.116				0.703
Ref: Police staff	1.00				1.00				1.00					1.00			
Police constable/sergeant	1.10	(0.87	1.40)		1.04	(0.79	1.38)		1.22	(0.95	1.56)			0.95	(0.71	1.26)	
Inspector/chief inspector or above	0.82	(0.38	1.78)		1.82	(0.79	4.26)		0.77	(0.52	1.14)			0.98	(0.63	1.53)	
Job strain				0.703				0.639				0.074					0.433
Ref: Low (high control, low demand)	1.00				1.00				1.00					1.00			
Passive (low control, low demand)	1.09	(0.81	1.45)		1.01	(0.72	1.42)		1.41	(1.08	1.84)			1.16	(0.88	1.59)	
Active (high demand, high control)	0.84	(0.61	1.16)		0.85	(0.60	1.22)		1.27	(0.99	1.61)			1.24	(0.97	1.79)	
High (high demand, low control)	1.34	(1.00	1.81)		1.13	(0.82	1.57)		1.66	(1.30	2.12)			1.38	(1.06	1.58)	
Work environment				–				–				–					–
Ref: Mainly office duties	1.00				1.00				1.00					1.00			
Mainly mobile duties	1.26	(0.96	1.67)		0.83	(0.60	1.15)		1.71	(1.38	2.12)			1.16	(0.91	1.47)	
Physical activity/week				0.326				0.181				0.910					0.808
Ref: Low	1.00				1.00				1.00					1.00			
Moderate	0.86	(0.63	1.16)		0.80	(0.58	1.10)		1.02	(0.76	1.35)			1.04	(0.77	1.40)	
High	0.51	(0.37	0.72)		0.48	(0.34	0.69)		0.58	(0.43	0.78)			0.60	(0.44	0.82)	
Smoking status				0.068				0.037				0.150					0.997
Ref: Never smoker	1.00				1.00				1.00					1.00			
Former smoker	1.29	(0.98	1.69)		1.37	(1.02	1.83)		0.85	(0.67	1.06)			1.00	(0.79	1.27)	
Current smoker	3.35	(2.47	4.55)		3.07	(2.19	4.29)		1.9	(1.41	2.58)			1.71	(1.23	2.37)	
Sleep				0.203				0.288				0.245					0.562
5 h or less	1.00				1.00				1.00					1.00			
6 h	0.98	(0.60	1.61)		0.92	(0.54	1.58)		0.80	(0.54	1.20)			0.81	(0.52	1.24)	
7 h	0.98	(0.61	1.55)		0.99	(0.60	1.64)		0.74	(0.50	1.09)			0.69	(0.45	1.06)	
8 h	0.98	(0.61	1.59)		0.81	(0.48	1.38)		0.73	(0.48	1.12)			0.69	(0.44	1.09)	
9 h or more	1.92	(1.04	3.56)		1.6	(0.82	3.13)		0.85	(0.44	1.61)			0.61	(0.30	1.23)	
Weekly hours sitting (weekdays)				0.708				0.760				0.356					0.533
Ref: Low (< 20 h)	1.00				1.00				1.00					1.00			
Moderate (20–40 h)	0.95	(0.73	1.23)		1.05	(0.78	1.39)		1.11	(0.89	1.37)			1.08	(0.86	1.35)	
High (> 40 h)	1.03	(0.78	1.36)		1.18	(0.86	1.62)		0.90	(0.71	1.13)			0.87	(0.68	1.12)	
TV viewing, hour per week				0.978				0.455				< 0.001					< 0.001
Ref: Low (< 6 h)	1.00				1.00				1.00					1.00			
Moderate (6–15 h)	1.00	(0.78	1.28)		1.11	(0.85	1.46)		1.51	(1.20	1.92)			1.65	(1.29	2.12)	
High (> 15 h)	1.17	(0.87	1.57)		1.27	(0.92	1.76)		1.57	(1.22	2.02)			1.74	(1.33	2.28)	

*GCSE* General Certificate of Secondary Education, *EI* energy intake

*P value for trend (region, work environment and marital status not calculated as no rank).

Women: ^a^397/2195 classified as recoding a ‘poor diet quality’, with exception of rank/work environment 342/1861 due to missing data against these variables (detailed in Table 1)

^b^Adjusted for age, education, household income, marital status, physical activity, region, smoking status, and daily TV viewing; 369/2099 classified as recording a ‘poor diet quality’ due to missing data, with exception of rank/work environment 321/1788 due to missing data against these variables (detailed in Table 1)

Men: ^c^590/3332 classified as recording a ‘poor diet quality’, with exception of rank/work environment 527/2865 due to missing data against these variables (detailed in Table 1)

^d^Adjusted for age, education, household income, marital status, physical activity, region, smoking status and daily TV viewing, 577/3267 classified as recording a ‘poor diet quality’ due to missing data, with exception of rank/work environment 518/2814 due to missing data against these variables (detailed in Table 1)

## Discussion

The present study demonstrated that diet quality determined by DASH score to be negatively associated with cardiometabolic risk within British police force employees. Employees in the lowest fifth of DASH score distribution had increased odds of cardiometabolic risk independent of other lifestyle factors and BMI. This study has also identified that a poor dietary score was also associated with other negative lifestyle behaviours (inactivity, smoking and TV viewing). Furthermore, employees reporting long working hours and high job strain (for men) had increased odds of reporting a dietary intake that was associated with cardiometabolic risk.

Previous studies in German and US police officers have observed the prevalence of metabolic syndrome to be higher in this occupational group compared to the general population [24, 25]. In the present study, two-fifths of male police force employees had three or more cardiometabolic risk factors. This is higher than the estimated prevalence of metabolic syndrome for the general European population of ~ 20–30% [26]. However, the prevalence amongst women in the present study was comparable to those reported in the general European population. The difference in the prevalence of increased metabolic risk may be due to the criteria applied in the present study (excluding triglycerides as unavailable and inclusion of CRP). Although the cardiometabolic scoring system used in the present study has not been validated to predict future cardiometabolic disease, it contains markers of risk (non-HDL and Hs-CRP) previously independently associated with cardiometabolic risk, and therefore, addresses the limitations of the metabolic syndrome definition previously cited by The CardioMetabolic Health Alliance [27].

There were clear dietary differences across men and women, with female police employees more likely to report a diet with a higher concentration of fruit and vegetables, and lower concentration of processed and red meat. This observation supports a previous study that also reported that women more likely make healthier choices (higher fruit and vegetable intake with lower intake of high fat foods) compared to men [28]. The differences in dietary intakes between men and women are likely to be multifactorial [28], but within the Airwave Health Monitoring Study cohort may relate to shorter working hours or different job roles allowing female employees more control over dietary choices.

Despite DASH being commonly applied to US cohorts and its recommendation by the American Heart Association, to date the DASH score has not been widely applied to UK cohorts to determine cardiometabolic health [11]. In the present study, classification of reporting a poorer quality dietary pattern (being in the lowest fifth for DASH score) was characterised by a total fat, saturated fat and NME intake above UK dietary guidelines, high intakes of alcohol, and less than two portions of fruit and vegetables per day (Supplementary Table S3). The present study found that those in the lowest quintile of DASH score had over double the odds of having three or more metabolic risk markers compared to those in the highest DASH score category (age adjusted). The relationship across DASH score groups showed a dose–response with only slight attenuation in the strength of association after adjusting for other lifestyle factors (smoking, physical activity, TV viewing, energy intake) and BMI. The observation that DASH score was negatively associated with having three or more cardiometabolic risk markers is in agreement with a previous study in Iranian nurses that found participants in the highest DASH score tertile had 81% lower odds of metabolic syndrome [29]. In conjunction with a recent trial in the UK conferring the DASH diet adaptability and acceptability to a UK population [30] our observations suggest that the DASH diet could provide an effective intervention to improve cardiometabolic risk profile of police force employees and potentially the general UK population.

In agreement with previous studies in European populations, we observed advancing age [31] and higher education [32] to be negatively associated with poorer quality dietary intakes. We found no association between police rank and recording a poor quality diet. This finding does not reflect observations from the Whitehall II Study that reported employment grade to be associated with differences in dietary behaviours, with those in a lower employment grade consuming a poorer diet [33]. A possible explanation is that, within police employment ranks there is more heterogeneity in terms of job role and working environment compared to predominantly office-based civil servants. The geographical differences in dietary intakes were observed across regions of employment in the Airwave Health Monitoring Study largely reflect those previously reported [34]. Comprehensive analyses of British dietary intakes have identified populations in Scotland and Wales as consuming diets higher in saturated fat and sodium, and lower in fruit and vegetables compared to the English population [34]. The association between low DASH score and low physical activity and current smoking status are suggestive of an overall clustering unhealthy lifestyle behaviours, which have been reported previously [35]. A small cross-sectional study in police officers in Pennsylvania, USA (*n* = 247) observed that fruit and vegetable intake was positively associated with physical activity [36]. In the present study TV viewing, but not weekday sitting, was associated with a poor diet quality in men. The Nurses’ Health Study (female only) similarly found that higher weekly TV viewing was associated with a diet high in energy, saturated fats, snacks and sweets [37].

We observed that the number of weekly working hours had a positive association with odds of recording a poor quality diet a relationship that was independent of age, education and region of employment for men. Previous studies investigating dietary intake and number of working hours have mainly used food frequency questionnaires or food surveys to collect dietary data [38–40], therefore, it is difficult to directly compare the results from the Airwave Health Monitoring Study. In general, our findings support the existing research that shows employees working longer weekly hours have higher intakes of take-away foods, convenience foods and snacks [38–40], which are indicative of a poorer quality diet. Job strain in men was also associated with diet quality, independent of established predictors. Previous research has shown psychological job demands to be positively associated with high fat food intakes in men, but not women [41]. High job strain is associated with longer working hours in the Airwave Health Monitoring Study Cohort. To determine if job strain contributed to the association between long hours and poorer diet quality in men exploratory analyses were conducted that repeated the multivariable logistic regression with both job strain and working hours (data not presented). Following this additional adjustment, long working hours (≥ 49 h per week) remained significantly associated with recording a poorer quality diet.

The strengths of the present study are the large-scale collection of 7-day estimated weighted diet records from a young single occupational group who are potentially at higher risk of cardiometabolic disease compared to the general population [24, 25]. The benefit of prospective measurement methods such as diet diaries, compared to food frequency questionnaires is that they do not measure against a predefined food list and allow more detailed dietary intake to be captured within occupational cohorts [42]. Additionally, food diaries do not rely on participant memory and recall ability. Additionally, the application of the DASH score to a UK population has been previously limited [11], therefore, these results strengthen the case for exploring the application of DASH diet guidelines as an intervention in the UK population. Our study has a number of limitations. Limited self-reported shift work information is currently only available for 12% of the cohort, and therefore, not included in the present study. The Buffalo Cardio-Metabolic Occupational Police Stress Study did observe an association between shift work and poor diet quality (measured by Dietary Inflammatory Index score) [43]. In the Airwave Health Monitoring Study cohort preliminary analyses have shown shift work prevalence is significantly associated with duration of weekly working hours (i.e., employees who are in the highest groups of weekly working hours are more likely to undertake shift work). Unfortunately, the interaction between shift work and long-working hours on determining dietary quality could not be explored due to the small sample size available. As with all current dietary recording tools, bias in reporting is an acknowledged limitation in nutritional research. Based on the food diary data, the estimated prevalence of misreporting energy intake was 50% in the Airwave Health Monitoring Study, a figure that is comparable to that reported in studies of the general UK population [17]. We, therefore, conducted sensitivity analyses excluding those classed as under-reporting energy intake (data not presented). From these analyses, we observed the significant association between diet quality and high cardiometabolic risk was lost for men after adjustment for BMI, a finding that would be expected given that BMI is strongly associated with under-reporting in the Airwave Health Monitoring Study Cohort [17]. The cross-sectional design of this study cannot infer a temporal pathway between long work hours or job strain and deterioration in dietary quality. Our observations may also be subject to residual confounding, as information on existing work place health initiatives was not collected as part of the study, this may be important to ascertain in future studies given that the Food Choice at Work Study in Ireland found employees with higher nutritional knowledge had a higher DASH score [44].

In conclusion, this study has profiled the dietary intakes and measured the diet quality (using the DASH score) of British police force employees. The novel aspect to this study has been the application of 7-day food diaries in the investigation of occupational exposures and diet quality. In general, the observed differences in diet quality across different employee characteristics (sex, region and age) in the police force reflect those within the general British population. Our findings suggest that for male police force employees longer duration of weekly working hours and higher job strain are associated with reporting a diet quality indicative of increased cardiometabolic risk. These findings potentially support the suggestion that dietary differences may contribute to variation in cardiometabolic disease risk observed across employees working longer hours or with high job strain. To test this hypothesis, future longitudinal studies are required to investigate if diet quality meditates the relationship between these occupational exposures and cardiometabolic disease. Additionally, further investigation to examine the barriers to healthy eating amongst male employees working extended hours or with high job strain is warranted. Gaining an understanding of why this cohort of employees are more likely to consume a diet associated with increased cardiometabolic risk will assist nutritional and occupational practitioners in targeting workplace nutritional interventions.

## Electronic supplementary material

Below is the link to the electronic supplementary material.

Supplementary material 1 (DOCX 71 KB)

## References

[1] World Health Organization (1985). Identification and control of work-related diseases.

[2] Gan Y, Yang C, Tong X, Sun H, Cong Y, Yin X (2014). Shift work and diabetes mellitus: a meta-analysis of observational studies. Occup Environ Med.

[3] Kivimäki M, Jokela M, Nyberg ST, Singh-Manoux A, Fransson EI, Alfredsson L (2015). Long working hours and risk of coronary heart disease and stroke: a systematic review and meta-analysis of published and unpublished data for 603 838 individuals. Lancet.

[4] Chandola T, Brunner E, Marmot M (2006). Chronic stress at work and the metabolic syndrome: prospective study. BMJ.

[5] Department of Health (2014) NHS Choices Boost your health at work. http://www.nhs.uk/Livewell/workplacehealth/Pages/workplaceoverview.aspx Accessed 5 Jan 2015

[6] Ezzati M, Riboli E (2013). Behavioral and Dietary Risk Factors for Noncommunicable Diseases. N Engl J Med.

[7] Dyson P. A., Kelly T., Deakin T., Duncan A., Frost G., Harrison Z., Khatri D., Kunka D., McArdle P., Mellor D., Oliver L., Worth J. (2011). Diabetes UK evidence-based nutrition guidelines for the prevention and management of diabetes. Diabetic Medicine.

[8] National Institute for Health and Care Excellence (2015) Maintaining a healthy weight and preventing excess weight gain among adults and children. NICE guidelines NG7

[9] Calton EK, James AP, Pannu PK, Soares MJ (2014). Certain dietary patterns are beneficial for the metabolic syndrome: reviewing the evidence. Nutr Res.

[10] de Koning L, Chiuve SE, Fung TT, Willett WC, Rimm EB, Hu FB (2011). Diet-quality scores and the risk of type 2 diabetes in men. Diabetes Care.

[11] Siervo M, Lara J, Chowdhury S, Ashor A, Oggioni C, Mathers JC (2015). Effects of the dietary approach to stop hypertension (DASH) diet on cardiovascular risk factors: a systematic review and meta-analysis. Br J Nutr.

[12] Fung TT, Chiuve SE, McCullough ML, Rexrode KM, Logroscino G, Hu FB (2008). Adherence to a DASH-style diet and risk of coronary heart disease and stroke in women. Arch Intern Med.

[13] Ball K, Timperio AF, Crawford DA (2006). Understanding environmental influences on nutrition and physical activity behaviors: where should we look and what should we count?. Int J Behav Nutr Phys Act.

[14] Association of Chief Police Officers (2012). UK Police Directory 2012.

[15] Elliott P, Vergnaud A-C, Singh D, Neasham D, Spear J, Heard A (2014). The Airwave Health Monitoring Study of police officers and staff in Great Britain: Rationale, design and methods. Environ Res.

[16] Nelson M, Haraldsdottir J (1998). Food photographs: practical guidelines: development and use of photographic atlases for assessing food portion size. Pub Health Nutr.

[17] Gibson Rachel, Eriksen Rebeca, Lamb Kathryn, McMeel Yvonne, Vergnaud Anne-Claire, Spear Jeanette, Aresu Maria, Chan Queenie, Elliott Paul, Frost Gary (2017). Dietary assessment of British police force employees: a description of diet record coding procedures and cross-sectional evaluation of dietary energy intake reporting (The Airwave Health Monitoring Study). BMJ Open.

[18] Black AE (2000). Critical evaluation of energy intake using the Goldberg cut-off for energy intake:basal metabolic rate. A practical guide to its calculation, use and limitations. Int J Obes Relat Metab Disord.

[19] Landsbergis PA, Schnall PL, Pickering TG, Schwartz JE (2002). Validity and reliability of a work history questionnaire derived from the Job Content Questionnaire. J Occup Environ Med.

[20] Ingre M (2015). Excuse me, but did the IPD-work consortium just “falsify” the job-strain model?. Scand J Work Environ Health.

[21] Craig CL, Marshall AL, Sjöström M, Bauman AE, Booth ML, Ainsworth BE (2003). International physical activity questionnaire: 12-country reliability and validity. Med Sci Sports Exerc.

[22] IPAQ scoring protocol (2005) http://www.ipaq.ki.se/scoring.htm Accessed 20 Oct 2014

[23] Beasley TM, Schumacker RE (1995). Multiple regression approach to analyzing contingency tables: Post hoc and planned comparison procedures. J Exp Educ.

[24] Anderson AA, Yoo H, Franke WD (2016). Associations of physical activity and obesity with the risk of developing the metabolic syndrome in law enforcement officers. J Occup Environ Med.

[25] Humbarger CD, Crouse SF, Womack JW, Green JS (2004). Frequency of metabolic syndrome in police officers compared to NCEP III prevalence values. Med Sci Sport Exerc.

[26] Grundy SM (2008). Metabolic syndrome pandemic. Arterioscler Thromb Vasc Biol.

[27] Sperling LS, Mechanick JI, Neeland IJ, Herrick CJ, Després J-P, Ndumele CE (2015). The cardioMetabolic health alliance: working toward a new care model for the metabolic syndrome. J Am Coll Cardiol.

[28] Wardle J, Haase AM, Steptoe A, Nillapun M, Jonwutiwes K, Bellisie F (2004). Gender differences in food choice: the contribution of health beliefs and dieting. Ann Behav Med.

[29] Saneei P, Fallahi E, Barak F, Ghasemifard N, Keshteli AH, Yazdannik AR (2015). Adherence to the DASH diet and prevalence of the metabolic syndrome among Iranian women. Eur J Nutr.

[30] Harnden KE, Frayn KN, Hodson L (2010). Dietary approaches to stop hypertension (DASH) diet: applicability and acceptability to a UK population. J Hum Nutr Diet.

[31] Alkerwi A, Vernier C, Crichton GE, Sauvageot N, Shivappa N, Hébert JR (2014). Cross-comparison of diet quality indices for predicting chronic disease risk: findings from the Observation of Cardiovascular Risk Factors in Luxembourg (ORISCAV-LUX) study. Br J Nutr.

[32] Finger JD, Tylleskär T, Lampert T, Mensink GBM, Irala-Estevez J, De Groth M (2013). Dietary behaviour and socioeconomic position: the role of physical activity patterns. PLoS One Pub Library Sci.

[33] Marmot MG, Stansfeld S, Patel C, North F, Head J, White I (1991). Health inequalities among British civil servants: the Whitehall II study. Lancet.

[34] Scarborough P, Morgan RD, Webster P, Rayner M (2011). Differences in coronary heart disease, stroke and cancer mortality rates between England, Wales, Scotland and Northern Ireland: the role of diet and nutrition. BMJ Open.

[35] Alkerwi A, Vernier C, Crichton GE, Sauvageot N, Shivappa N, Hébert JR (2014). Cross-comparison of diet quality indices for predicting chronic disease risk: findings from the Observation of Cardiovascular Risk Factors in Luxembourg (ORISCAV-LUX) study. Br J Nutr.

[36] Can SH, Hendy HM (2014). Behavioral variables associated with obesity in police officers. Ind Health.

[37] Hu FB, Li TY, Colditz GA, Willett WC, Manson JE (2003). Television watching and other sedentary behaviors in relation to risk of obesity and type 2 diabetes mellitus in women. JAMA.

[38] Fan W, Lam J, Moen P, Kelly E, King R, McHale S (2015). Constrained choices? Linking employees’ and spouses’ work time to health behaviors. Soc Sci Med.

[39] Escoto KH, Laska MN, Larson N, Neumark-Sztainer D, Hannan PJ (2012). Work hours and perceived time barriers to healthful eating among young adults. Am J Health Behav.

[40] Escoto KH, French SA, Harnack LJ, Toomey TL, Hannan PJ, Mitchell NR (2010). Work hours, weight status, and weight-related behaviors: a study of metro transit workers. Int J Behav Nutr Phys Act.

[41] Hellerstedt WL, Jeffery RW (1997). The association of job strain and health behaviours in men and women. Int J Epidemiol.

[42] Lindström J (2016). Does higher energy intake explain weight gain and increased metabolic risks among shift workers?. Scand J Work Environ Health.

[43] Wirth MD, Burch J, Shivappa N, Violanti JM, Burchfiel CM, Fekedulegn D (2014). Association of a dietary inflammatory index with inflammatory indices and metabolic syndrome among police officers. J Occup Environ Med.

[44] Geaney F, Fitzgerald S, Harrington JM, Kelly C, Greiner BA, Perry IJ (2015). Nutrition knowledge, diet quality and hypertension in a working population. Prev Med Rep.

