# Supplementary material for: A cross-sectional investigation into the occupational and socio-demographic characteristics of British police force employees reporting a dietary pattern associated with cardiometabolic risk: findings from the Airwave Health Monitoring Study
Source: Eur J Nutr. 2017 Nov 2;57(8):2913–26. doi: 10.1007/s00394-017-1562-4 (PMC6267403; doi:10.1007/s00394-017-1562-4)
Supplement: Supplementary file 1 — Supplementary material 1 (DOCX 71 KB) [file 394_2017_1562_MOESM1_ESM.docx]

**A cross-sectional investigation into the occupational and socio-demographic characteristics of British police force employees reporting a dietary pattern associated with cardiometabolic risk: Findings from the Airwave Health Monitoring Study**

**Authors** Rachel Gibson^1^, Rebeca Eriksen^1^, Deepa Singh^2^, Anne-Claire Vergnaud^2^, Andrew Heard^2^, Queenie Chan^2^, Paul Elliott^2^ and Gary Frost^1^.

**Table S1** Food group descriptions used for the DASH food groups

| **Food group** | **Items included** | **Items not included** |
| --- | --- | --- |
| Whole grains | Amaranth | Barley, pearled |
|  | Barley, whole, Barley meal | Barley malt flour |
|  | Brown rice, Brown rice flour, Wild rice | Bran (all grains) |
|  | Buckwheat | Corn flour/meal |
|  | Bulgur wheat | Couscous |
|  | Millett | Rice (milled, not whole grain) |
|  | Oats, Oat flour, Oatmeal | Semolina |
|  | Popcorn (only include plain) | Wheat flour (milled, not whole grain) |
|  | Quinoa | Brown flour |
|  | Whole wheat flour | Brown bread |
|  | Shredded wheat | Cornflakes |
|  | Dark rye bread / pumpernickel | Rice krispie |
|  | Muesli | Egg / rice / plain noodles |
|  |  |  |
|  | Ready Brek / quick cook oats | Crisp bread |
|  | Weetabix, Cheerios, Shreddies, All-bran | Bran flakes |
|  | Whole wheat pasta | Nutri-grain |
|  | Ryvita / rye crackers | Special K |
|  | Oatcakes | Granary bread |
|  | Whole wheat bread | Flapjack |
|  |  | Cakes, pastry, biscuits made with wholemeal flour |
| Low fat dairy | skimmed, semi skimmed milk, 1% fat milk, cottage cheese, low fat and fat free yoghurt | Dairy alternatives |
|  |  |  |
|  |  |  |
| Nuts, seeds, legumes | All nuts, peanuts | Seed oils |
|  | Beans | Green bean/ French bean |
|  | Lentils | Processed soya bean products |
|  |  |  |
|  | Chickpeas | Bean flours |
|  |  | Coconut |
|  |  | Marzipan |
| Fruit | Fresh fruit | Fruit in cakes and confectionary |
|  | Dried fruit | Jam / chutney |
|  | Pre prepared fruit including frozen and canned) | Fruit juices as cordial |
|  | 100% pure fruit juices |  |
| Vegetables | Sweet potato | White potato |
| (UK culinary usage classification) | All fresh, and prepared vegetables (frozen, dried, canned) | Cassava |
|  | Herbs | Chutney |
|  | Tomatoes |  |
| Red and processed meat | Beef, Lamb, Pork, Veal, Mutton, Horse, | Poultry |
|  | Goat, Venison, Game meat, Offal (from any animal). |  |
|  |  |  |
|  | Deli meats, such as sliced turkey and bologna, Bacon, Sausages/ Hot dogs/frankfurters, Ham, Pastrami Luncheon meats. |  |
| Sugar sweetened beverages | Cordials and fruit juices with added sugar | Low calorie or artificial sweetened beverages |
|  | Energy drinks | 100% pure fruit juices |
|  |  |  |
|  | Carbonated sweetened beverages (e.g. colas, lemonade) |  |
|  |  |  |

**Table S2** Quintile cut-offs for calculating DASH scores based on individuals from the Airwave Health Monitoring Study

| **DASH food groups** | **20%** | **40%** | **60%** | **80%** |
| --- | --- | --- | --- | --- |
| Whole grains g/day |  |  |  |  |
| men | 8.1 | 27.7 | 51.1 | 89.9 |
| women | 8.4 | 23.8 | 41.3 | 67.3 |
| Low fat dairy g/day |  |  |  |  |
| men | 88.7 | 156.6 | 220.0 | 302.6 |
| women | 72.1 | 129.0 | 190.1 | 269.9 |
| Nuts, seeds and legumes g/day |  |  |  |  |
| men | 9.6 | 19.0 | 29.4 | 44.7 |
| women | 7.4 | 16.7 | 25.8 | 41.0 |
| Fruits g/day |  |  |  |  |
| men | 42.9 | 102.1 | 167.7 | 266.0 |
| women | 47.7 | 100.2 | 160.0 | 251.5 |
| Vegetables (excl. potatoes) g/day |  |  |  |  |
| men | 72.4 | 105.2 | 138.4 | 185.0 |
| women | 80.2 | 112.4 | 148.9 | 200.1 |
| Processed and red meat g/day |  |  |  |  |
| men | 43.0 | 64.2 | 85.2 | 110.4 |
| women | 21.9 | 40.2 | 57.4 | 80.4 |
| Sugar sweetened beverages g/day |  |  |  |  |
| men | 0.0 | 28.6 | 98.0 | 274.0 |
| women | 0.0 | 29.4 | 92.9 | 233.3 |
| Sodium mg/day* |  |  |  |  |
| men | 2391.6 | 2798.7 | 3191.9 | 3696.0 |
| women | 1906.7 | 2242.9 | 2584.2 | 3008.4 |

*Sodium is includes only intrinsic sodium (not discretionary salt added to cooking and/or at the table)

**Table S3** Dietary profile by quintile cut off of DASH score for men and women in the Airwave Health Monitoring Study

|  | **DASH score group based on quintile cut off** | | | | | | | | | |  |
| --- | --- | --- | --- | --- | --- | --- | --- | --- | --- | --- | --- |
| **MEN** | **Q1**  **(least healthy)** | | **Q2** | | **Q3** | | **Q4** | | **Q5**  **(most healthy)** | | *p* |
| **DASH score median (range)** | 17 | 9 - 19 | 21 | 20 -22 | 24 | 23 - 25 | 27 | 26 - 28 | 31 | 29- 39 |  |
|  | Mean (SD) | | | | | | | | | |  |
| **Nutrient and food intake** |  |  |  |  |  |  |  |  |  |  |  |
| % energy intake – total fat | 35.9 | 0.2 | 34.5 | 0.2 | 34.1 | 0.2 | 32.4 | 0.2 | 30.9 | 0.2 | **<0.0001** |
| % energy intake – saturated fat | 13.5 | 0.1 | 12.9 | 0.1 | 16.7 | 0.1 | 11.8 | 0.1 | 10.8 | 0.1 | **<0.0001** |
| % energy intake – MUFA | 12.6 | 0.1 | 12.0 | 0.1 | 11.8 | 0.1 | 11.1 | 0.1 | 10.5 | 0.1 | **<0.0001** |
| % energy intake – PUFA | 5.9 | 0.1 | 5.7 | 0.1 | 5.8 | 0.1 | 5.6 | 0.1 | 5.7 | 0.1 | **0.009** |
| % energy intake - carbohydrate | 44.1 | 0.3 | 45.5 | 0.3 | 46.4 | 0.2 | 47.5 | 0.2 | 49.8 | 0.3 | **<0.0001** |
| % energy intake NME | 12.8 | 0.2 | 11.9 | 0.2 | 11.5 | 0.2 | 11 | 0.2 | 10.8 | 0.2 | **<0.0001** |
| % energy intake - protein | 16.5 | 0.1 | 16.9 | 0.1 | 17.0 | 0.1 | 17.4 | 0.1 | 17.9 | 0.1 | **<0.0001** |
| Energy density food kcal/g | 1.83 | 0.01 | 1.65 | 0.01 | 1.56 | 0.01 | 1.43 | 0.01 | 1.35 | 0.01 | **<0.0001** |
| NSP g/1000kcal | 4.9 | 0.1 | 5.8 | 0.1 | 6.7 | 0.1 | 7.5 | 0.1 | 8.9 | 0.1 | **<0.0001** |
| % energy intake – Alcohol | 5.0 | 9.1 | 4.6 | 8.5 | 4.0 | 6.9 | 4.7 | 7.2 | 3.6 | 6.0 | **0.0003** |
| Vegetables grams/1000kcal | 48.9 | 30.4 | 61.5 | 35.9 | 71.5 | 41 | 82.3 | 45.1 | 101.6 | 50.8 | **<0.0001** |
| Fruit grams/1000kcal | 22.2 | 43.1 | 43.7 | 59.1 | 63 | 68 | 87.9 | 77 | 123.7 | 93.7 | **<0.0001** |
| Wholegrain grams/1000kcal | 3.7 | 12.1 | 10.8 | 20 | 18.9 | 26.3 | 28.2 | 31.2 | 39.4 | 36.2 | **<0.0001** |
| Total dairy grams/1000kcal | 79.6 | 58.6 | 104.5 | 68.3 | 121.3 | 73.0 | 133.8 | 76.6 | 160.7 | 81.2 | **<0.0001** |
| Fish grams/1000kcal | 4.6 | 12.2 | 7.5 | 14.8 | 7.5 | 15.4 | 10.2 | 16.4 | 13.1 | 18.5 | **<0.0001** |
| Total red and processed meat g/1000kcal | 47.9 | 25.3 | 41.1 | 26.9 | 36.9 | 23.7 | 32.1 | 23.1 | 24.1 | 20.4 | **<0.0001** |
| Sugar sweetened beverages g/1000kcal | 99.5 | 169.2 | 49.3 | 125.7 | 31.2 | 94.4 | 15.3 | 56.7 | 0 | 24.7 | **<0.0001** |
| Sodium mg/1000kcal | 1570 | 12 | 1530 | 11 | 1506 | 10 | 1452 | 11 | 1387 | 11 | **<0.0001** |
|  |  | | | | | | | | | |  |
| Table S3 continued | **DASH score group based on quintile cut off** | | | | | | | | | |  |
| **WOMEN** | **Q1**  **(least healthy)** | | **Q2** | | **Q3** | | **Q4** | | **Q5**  **(most healthy)** | | *p* |
| **DASH score median (range)** | 18 | 9- 19 | 21 | 20- 22 | 24 | 23 - 25 | 27 | 26 - 28 | 31 | 29 -38 |  |
| **Nutrient and food intake** | Mean (SD)      Mean (SD) | | | | | | | | | |  |
| % energy intake – total fat | 35.9 | 0.3 | 34.6 | 0.3 | 34.2 | 0.2 | 32.9 | 0.2 | 31.5 | 0.3 | **<0.0001** |
| % energy intake – saturated fat | 13.4 | 0.1 | 12.9 | 0.1 | 12.6 | 0.1 | 12 | 0.1 | 11.1 | 0.1 | **<0.0001** |
| % energy intake – MUFA | 12.5 | 0.1 | 11.8 | 0.1 | 11.6 | 0.1 | 11 | 0.1 | 10.5 | 0.1 | **<0.0001** |
| % energy intake – PUFA | 6.1 | 0.1 | 5.9 | 0.1 | 6.0 | 0.1 | 5.9 | 0.1 | 5.9 | 0.1 | **0.413** |
| % energy intake - carbohydrate | 45.4 | 0.3 | 47.3 | 0.3 | 47.3 | 0.3 | 48.8 | 0.3 | 51.3 | 0.3 | **<0.0001** |
| % energy intake NME | 13.7 | 0.2 | 12.8 | 0.2 | 11.9 | 0.2 | 11.5 | 0.2 | 11.4 | 0.2 | **<0.0001** |
| % energy intake - protein | 16.3 | 0.2 | 16.6 | 0.2 | 17 | 0.1 | 17.4 | 0.2 | 17.4 | 0.2 | **<0.0001** |
| Energy density food kcal/g | 1.70 | 0.02 | 1.58 | 0.02 | 1.45 | 0.02 | 1.36 | 0.02 | 1.24 | 0.02 | **<0.0001** |
| NSP g/1000kcal | 5.3 | 0.1 | 6.4 | 0.1 | 7.2 | 0.1 | 8.3 | 0.1 | 9.7 | 0.1 | **<0.0001** |
| % energy intake – Alcohol | 4 | 7.2 | 3.1 | 6.8 | 3.2 | 6.7 | 2.9 | 6.5 | 2.3 | 5.1 | **<0.0001** |
| Vegetables grams/1000kcal | 60.6 | 39.2 | 78 | 47.7 | 92.6 | 54.1 | 112 | 60.6 | 130.9 | 80.6 | **<0.0001** |
| Fruit grams/1000kcal | 30.6 | 54.1 | 59.1 | 47.6 | 78.1 | 80.2 | 101 | 89.1 | 149.3 | 116.8 | **<0.0001** |
| Wholegrain grams/1000kcal | 4.8 | 13.4 | 13 | 20.4 | 19 | 26.5 | 26.8 | 27 | 36.1 | 30.1 | **<0.0001** |
| Total dairy grams/1000kcal | 78.5 | 64 | 109 | 76.3 | 124.5 | 83.1 | 149.1 | 90.9 | 184 | 107.3 | **<0.0001** |
| Fish grams/1000kcal | 6.5 | 13.1 | 7.9 | 16.2 | 9.4 | 19.7 | 12.5 | 19.8 | 13.5 | 22.1 | **<0.0001** |
| Total red and processed meat g/1000kcal | 40.3 | 26.3 | 35.4 | 25.8 | 30.6 | 26.3 | 24.7 | 25.9 | 16.3 | 22.8 | **<0.0001** |
| Sugar sweetened beverages g/1000kcal | 117.6 | 176.1 | 55.2 | 124.6 | 31 | 107.1 | 18.1 | 66.7 | 0 | 34.7 | **<0.0001** |
| Sodium mg/1000kcal | 1543 | 16 | 1517 | 15 | 1466 | 14 | 1495 | 14 | 1397 | 15 | **<0.0001** |

Abbreviations: EI energy intake, SD standard deviation, MUFA mono unsaturated fatty acids, NSP non-starch polysaccharides, PUFA polyunsaturated fatty acids. General linear model applied to test linear relationship between dietary intake across fifths of DASH score.
